# Computational Modelling and Clinical Validation of an Alzheimer’s-Related Network in Brain Cancer: The SKM034 Model

**DOI:** 10.3390/cimb48020126

**Published:** 2026-01-23

**Authors:** Kristy Montalbo, Izabela Stasik, Christopher George Severin Smith, Emyr Yosef Bakker

**Affiliations:** 1 School of Pharmacy and Biomedical Science, University of Lancashire, Preston PR1 2HE, UK; kmontalbo@lancashire.ac.uk (K.M.); istasik@lancashire.ac.uk (I.S.); 2 School of Medicine and Dentistry, University of Lancashire, Preston PR1 2HE, UK

**Keywords:** Alzheimer’s disease, glioblastoma, SORL1, Boolean model, clinical validation, network modelling

## Abstract

Cancer and Alzheimer’s disease (AD) display an inverse relationship, and there is a need to further explore this interplay. One key genetic contributor to AD is *SORL1*, the loss of which is thought to be causally related to AD development. SORL1 also appears to be implicated in cancer. To examine SORL1 and its network, this article simulated SORL1 and its interactions via signal-flow Boolean modelling, including in silico knockouts (mirroring in vivo loss-of-function mutations). This model (SKM034) predicted a total of 29 key changes in molecular relationships following the loss of SORL1 or another highly connected protein (ERBB2). Literature validation demonstrated that 2 of these predictions were at least partially validated experimentally, whilst 27 were Potentially Novel Predictions (PNPs). Complementing the in-depth relationship analyses was signal flow analysis through the network’s structure, validated using cell line and cancer patient RNA-seq data. Correct prediction rates for these analyses reached 60% (statistically significant relative to a random model). This article demonstrates the clinical relevance of this Alzheimer’s-related network in a cancer context and, through the PNPs, provides a strong starting point for in vitro experimental validation. As with previously published models using similar methods, the model may be reanalysed in different contexts for further discoveries.

## 1. Introduction

Alzheimer’s disease and cancer are diseases that disproportionately affect the elderly, with approximately 60% of cancer diagnoses occurring in those over 65 [1], whilst one of the biggest risk factors for Alzheimer’s disease is age [2]. Alzheimer’s disease is also the cause of between 60% and 80% of dementia cases [2]. The mortality rate of both diseases also increases with age [3].

Despite the above demographic similarities, the relationship between the two is unclear. Some argue that they are positively associated (e.g., findings of a positive correlation between the mortality rates of brain tumours and Alzheimer’s disease [3]), while other studies have suggested an inverse relationship, with one systematic review and meta-analysis concluding that the inverse association between Alzheimer’s disease and cancer was not seemingly a consequence of diagnostic bias, competing risks, or known confounders [4]. Other studies have found that gliomas and glioblastomas were associated with a significantly lower mortality risk for patients with Alzheimer’s disease compared to other tumours [5].

Given the potentially conflicting data and the importance of both diseases to the elderly population, further work is needed to understand their interplay. Alzheimer’s disease is known to have a significant genetic component (recently comprehensively reviewed in [6]). One of the most significant molecular drivers of Alzheimer’s disease is APP, or the amyloid precursor protein. APP is the precursor to the amyloid-beta peptide (Aβ peptide), which is known to accumulate in individuals with Alzheimer’s disease [7] and is thought to be central to its pathology. Processing and secretion of the Aβ peptide is controlled at several levels, but a key protein involved in this is SorLA (encoded by *SORL1*) [8]. Loss of *SORL1* is thought to be causally related to Alzheimer’s disease [9], and yet SORL1 is demonstrated to have increased expression in lymphoma [10] and has also been implicated in breast cancer [11].

Given the importance of SORL1 in both Alzheimer’s disease and cancer, the purpose of this study was to generate a computational mechanistic model of SORL1 and its interaction partners, with these partners being connected by activation or inhibition reactions. Such modelling has been historically employed in a cancer context, both for TP53 [12] and the glucocorticoid receptor (GR) [13]. These models demonstrated significant predictive utility for in silico simulations of in vivo loss-of-function mutations across a range of cancer cell lines [12,13], as well as in patient data on leukaemia [13] and mesothelioma [14]. This article outlines the development of a SORL1 model using a similar methodology—the SKM034 model (the SORL1 model by Kristy Montalbo, consisting of 34 nodes). The 34 nodes in the SKM034 model represent genes/proteins, and the nodes are connected by 92 interactions (edges) of activation or inhibition. The model has undergone permutation through in silico knockout simulations and demonstrated statistically significant correct prediction rates in both cell line and patient data. This article demonstrates the first successful application of this modelling approach to SORL1 biology and identifies areas for further research.

## 2. Materials and Methods

### 2.1. Model Construction: Extraction and Double Curation of STRING Data

The Search Tool for the Retrieval of Interacting Genes/Proteins (STRING) database v12.0 [15] was used as the initial starting point of protein interactions. This database was used to build the aforementioned TP53 and GR computational models [12,13]. Model construction proceeded in two phases: in phase one of model construction, proteins that had direct functional relationships with SORL1 were extracted and curated (see below). This formed the Primary Layer of the model. In phase two of the model construction, interactions between the SORL1-interacting proteins were extracted and curated. This formed the Second Layer of the model.

In the construction of the Second Layer, an “intermediary rule” was applied. This meant that if STRING predicted an interaction between Protein 1 and Protein 2 (both of which interacted with SORL1 individually) but literature showed that the relationship was actually via an intermediary (i.e., Protein 1 -> Intermediary Protein -> Protein 2), then how the interaction was logged was contingent on the status of the Intermediary Protein. If the Intermediary Protein was in the model, then the reactions would be logged as Protein 1 -> Intermediary Protein -> Protein 2, provided that there was no additional evidence supporting a direct relationship (in which case, both the direct relationship and indirect relationship would be added). If the Intermediary Protein was not in the model, then the interaction was instead put directly as Protein 1 -> Protein 2 in an effort to reduce redundancy.

Extraction of the Primary Layer began with the “9606.protein.links.v12.0.txt.gz” file on STRING. This contains all of STRING’s records of the links between different human proteins, alongside a confidence score. The STRING ID of SORL1 for *Homo sapiens* (9606.ENSP00000260197) was used to extract all interactions for SORL1, which were then filtered to include only interactions that were medium confidence or above (≥0.4/400). This final confidence threshold for inclusion was based on an iterative process during curation to reach a reasonable number of included interactants and interactions.

Curation of the Primary Layer then took place by examining the available evidence from STRING to find peer-reviewed articles that demonstrated a functional relationship of activation or inhibition between the two proteins it said were linked. Manual curation has been demonstrated to be an essential process when building mechanistic models due to the known issue of false positives due to, e.g., text mining errors in STRING [12,13]. In addition to looking at STRING, noting that v12.0 (the current version) was dated as current from 26 July 2023, additional literature searches were conducted on PubMed with the following search term:

(((Protein 1)) AND ((Protein 2))) AND ((“2023/07/01”[Date—Publication]: “2024/07/31”[Date—Publication]))

For example:

(((LPL OR LIPD OR HDLCQ11)) AND ((APOE OR AD2 OR APO-E OR ApoE4 OR LDLCQ5 OR LP))) AND ((“2023/07/01”[Date—Publication]: “2024/07/31”[Date—Publication]))

The date range of July 2023–July 2024 helped capture additional papers and interaction evidence published since STRING’s last update up to the point at which curation began. Interactions required evidence from at least one PubMed-indexed paper to be included in the model.

Following curation of the Primary Layer, “9606.protein.links.v12.0.txt.gz” was again accessed, and all interactions for all SORL1 interactants were extracted. This list of interactants was then filtered to include only interactions between genes/proteins present in the Primary Layer. The curation of the Second Layer proceeded as with the Primary Layer, with the additional requirement that if a significantly large number of PubMed papers were returned during the search for papers from 2023 to 2024, a filtration process was performed to reduce the number of articles required to screen, as summarised in Table 1.

All curations in both the Primary and Second Layer were performed in the first instance by author KM and then validated by either author IS, CGSS, or EYB to reinforce the integrity of the model. It was a curation requirement that the interaction record was both in a human context and of a brain-related biological origin. This helped ensure focus at both a species- and tissue-type level.

Once all interactions had been double-curated, the records were collated and imported into Cytoscape v3.10.3 [16] for model visualisation. Nodes of the model were considered the genes/proteins, whilst the edges were the interactions between the nodes. Based on the node number, the SORL1 model was deemed the SKM034 model (SORL1 model by Kristy Montalbo, consisting of 34 nodes). All interaction records, alongside PubMed ID evidence for them, can be found in Supplementary Table S1A,B of Supplementary File S1.

### 2.2. Model Analysis: CellNetAnalyzer

Once the model had been built, interaction records were converted into a format readable by CellNetAnalyzer and imported. CellNetAnalyzer (CNA) is a MATLAB (R2025a) toolbox which facilitates the analysis of both signal-flow and mass-flow (metabolic) networks [17]. Two analyses are predominant in CNA: logical steady state analysis (LSSA) and dependency matrices (DMs).

With LSSA, the goal is to determine the overall activation state (in terms of ON [1] or OFF [0]) of every node given a particular input. It is also possible for nodes to have a value of “NaN” (undetermined) if the node exhibits oscillatory behaviour or if there is insufficient information to determine its state. In terms of “levels of activation”, 1 is considered more active than NaN, which in turn is considered more active than 0. By default, all nodes are considered “NaN”; inputs are then set, and then analysis can be undertaken to follow the signal flow. For LSSA of the SKM034 model, two scenarios were used: SORL1 ON (1), which mirrors wild-type cells, and SORL1 OFF (0), which mirrors knockout (KO) cells.

Comparison of two LSSA scenarios permitted the calculation of *Emod*, i.e., the model’s prediction of whether individual nodes were up, down, or unchanged. A summary of how *Emod* was calculated is shown in Table 2.

As described in Section 2.3 and Section 2.4, the *Emod* values were compared to the actual changes observed in cell line or patient data (*Eexp*). This allowed for the calculation of the model’s accuracy.

Dependency matrix analysis permits an exhaustive search of all possible signalling paths and feedback loops within the model. Effectively, it allows for the determination of the overall effect *of* every node in the model *on* every node in the model. Based on the signalling paths between two nodes, six possible dependencies are possible, as summarised in Table 3.

Calculation of a DM for both wild-type and knockout scenarios and comparison of the two permitted the identification of granular, gene-specific changes that occur in knockout scenarios. These predictions could then be validated through literature searches or identified as a Potentially Novel Prediction (PNP) that may be validated experimentally [12,13].

### 2.3. Model Validation: Cell Line Data

As explained above, model validation is possible on both the LSS results and the changes identified from DM analyses. The SKM034 model was validated using both cell line and patient data.

A previous publication by Lee and colleagues [18] investigated the impact of *SORL1* KO (through CRISPR) across a range of brain cell types. Induced pluripotent stem cells (iPSCs) were differentiated to neuron, astrocyte, microglial, and endothelial cell fates for both *SORL1* KO and matched control cells, after which RNA-seq was performed. These experimental conditions mirrored the simulation analyses performed on the SORL1 model and were thus an excellent source to validate the model’s predictions. Supplementary Table S1 from Lee and colleagues [18] was accessed to obtain the RNA-seq expression matrix, which underwent additional analysis to generate *Eexp*.

Initially, *Eexp* was calculated solely on the *q*-value in the RNA-seq data and the direction of the fold change. This was performed through bespoke analysis of the RNA-seq data from Lee and colleagues [18]. The RNA-seq matrix included the replicate values (e.g., 4xWT and 4x*SORL1*-KO datasets), and so the first step was averaging these to a single value per experimental condition (WT and KO) on a cell line-by-cell line basis. Fold changes were then calculated by dividing the KO value by the WT value. *q*-values were then retrieved for each gene from the original supplementary file from Lee and colleagues [18]. The criteria in Table 4 were then used to generate the *Eexp*. Directionality of the FC was validated in each case through SORL1; as it was knocked out, it should have an *Eexp* of −1.

The absolute value of *Emod-Eexp* could then give rise to three possible values: 0, 1, or 2. A value of 0 meant that *Emod* and *Eexp* were the same, meaning the model’s prediction was correct. A value of 1 meant that *Emod* and *Eexp* were slightly different, e.g., the model predicted it was unchanged, but it went up or down. This was a “one-step” error and considered a “small error”. If the value was 2, this meant the model predicted the opposite of what occurred in reality (a “two-step” error) and was considered a “large error”. With three possible outcomes, a random model would achieve 1/3 predictions correct; thus, the *p*-value of the correct prediction rate was assessed using Excel’s BINOM.DIST formula, with the number of correct predictions as number_s, the number of nodes examined as trials, 1/3 as probability_s, and FALSE for cumulative. It should be noted that for all correct prediction rate calculations, SORL1 was excluded as it was the criterion rather than a result; its inclusion would otherwise have skewed the correct prediction rate upwards.

To assess the impact of the level of *SORL1* knockout on correct prediction rates, correct prediction rates per cell type were collated along with the FC calculated for SORL1. Pearson correlation was then performed, alongside the calculation of R^2^ and the *p*-value of the correlation (using the T-Distribution and T.DIST).

Building further on the above validation was the consideration of the level of biological change, rather than just *q*-value and FC < 1 or FC > 1. Additional analyses included filtration based on FC 1.5 or equivalent, FC 2 or equivalent, and FC 3 or equivalent (equivalent, in all cases, meaning the same proportional change in terms of downregulation rather than upregulation). This meant that even if the *q*-value was <0.05, the *Eexp* would still be 0 if the FC was beneath the biological threshold identified above. The same approach was then used to calculate the correct number of predictions as well as the *p*-value of the correct prediction rate.

### 2.4. Model Validation: Clinical Data

In addition to the cell line validation above, cBioPortal [19,20,21] was accessed to validate the model’s predictions at both the clinical (patient) level and in a cancer context. Six studies overall were used for this: Brain Lower Grade Glioma (TCGA, Firehose Legacy); Brain Lower Grade Glioma (TCGA, PanCancer Atlas); Brain Tumor PDXs (Mayo Clinic, Clin Cancer Res 2020); Glioblastoma (TCGA, Cell 2013); Glioblastoma Multiforme (TCGA, Firehose Legacy), and Glioblastoma Multiforme (TCGA, PanCancer Atlas).

Initially, on a per-study basis, mRNA expression z-scores relative to diploid samples were extracted from cBioPortal for all patients for SORL1. Four different analyses were performed on the basis of their z-scores: division by mean, division by median, division by upper quartile, and division by lower quartile. For each analysis and study in turn, groups were created outside of cBioPortal on the basis of the Sample ID, and then the Sample IDs were uploaded to cBioPortal to create custom groups of “High SORL1” and “Low SORL1”. Through cBioPortal’s Compare function, differential gene expression analysis was then performed. These data were then downloaded and analysed outside of cBioPortal. Initial *Eexp* calculation was based on the *q*-value and Log10 FC (Table 5). Directionality of the Log10 FC was validated in each case through SORL1; as it was the divisor between patients, it should have an *Eexp* of −1.

As with the cell line validation described above, the correct prediction rates were calculated using the absolute value of *Emod-Eexp,* and the *p*-value was calculated using Excel’s BINOM.DIST, as previously described.

Additionally, again, like the cell line validation above, the degree of biological change was captured through additional analyses looking at FC 1.5 or above, FC 2 or above, and FC 3 or above. These analyses were based on mRNA expression (RNA Seq V2 RSEM) rather than z-scores, and patients were again divided, in turn, by mean, median, upper quartile, and lower quartile. Correct prediction rates and *p*-values were calculated for each analysis as previously described.

## 3. Results

### 3.1. SORL1 Model Construction and Validation Workflow

**Figure 1:**
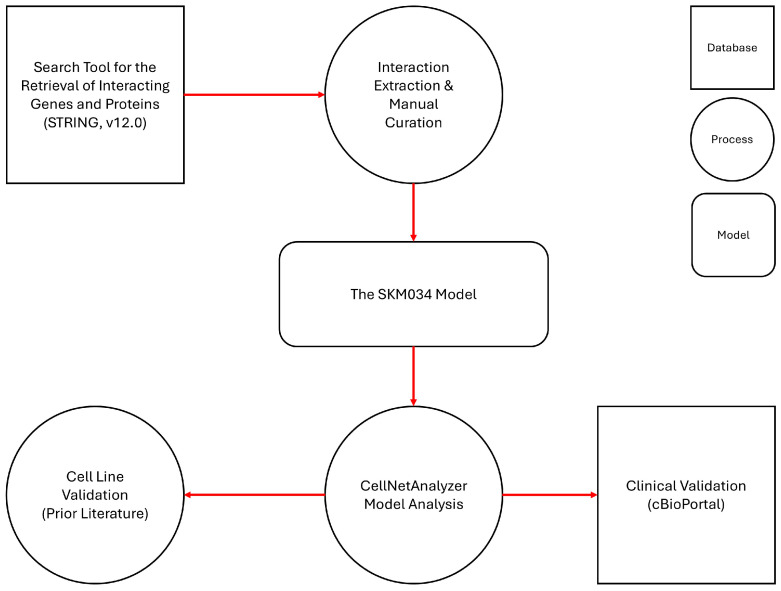
SKM034 construction and validation workflow. STRING represents the starting point of the model construction as it is the source of protein links, which are then double-curated. Protein links are captured for both SORL1 (as the central node) and links between the genes/proteins that interact with SORL1. These curated interaction records together form the SKM034 model, which is analysed in CellNetAnalyzer (CNA) and validated through both cell line data (from previously published literature) and at the clinical level using cancer patient data (from cBioPortal).

Section 2 describes the construction and validation of the SKM034 model in significantly more detail; however, the overall workflow is shown in Figure 1.

### 3.2. SKM034 Model Structure and Target Identification

**Figure 2:**
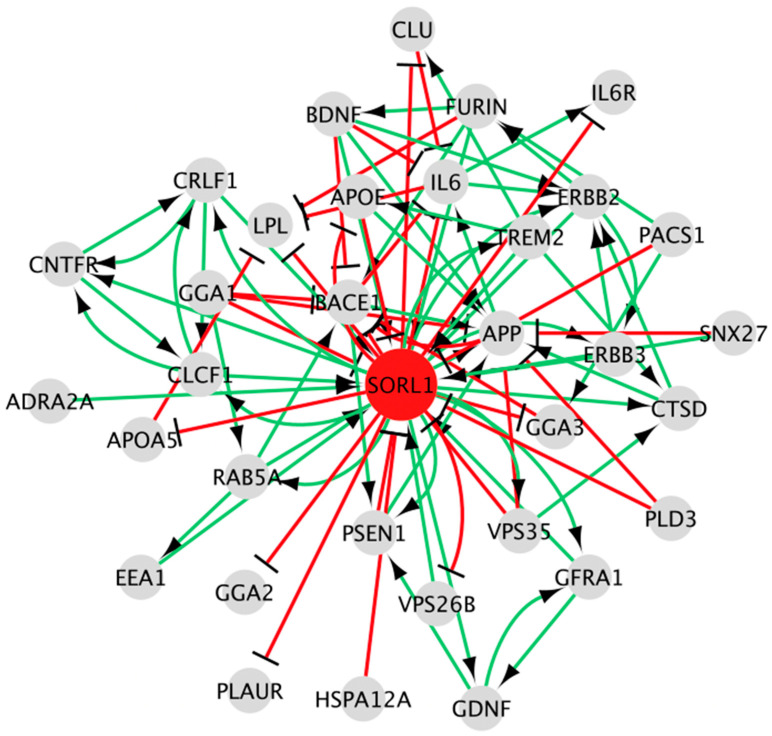
The SKM034 model. The central node (SORL1) is shown in red, whilst surrounding nodes (representing other genes/proteins) are shown in grey. The model consists of 34 nodes connected by 92 edges of either activation or inhibition. Green directed edges represent activation relationships, whilst red blunted edges represent inhibition relationships.

Following the workflow, the SKM034 model was developed, consisting of 34 nodes connected by 92 logical interactions of activation or inhibition (Figure 2).

**Figure 3:**
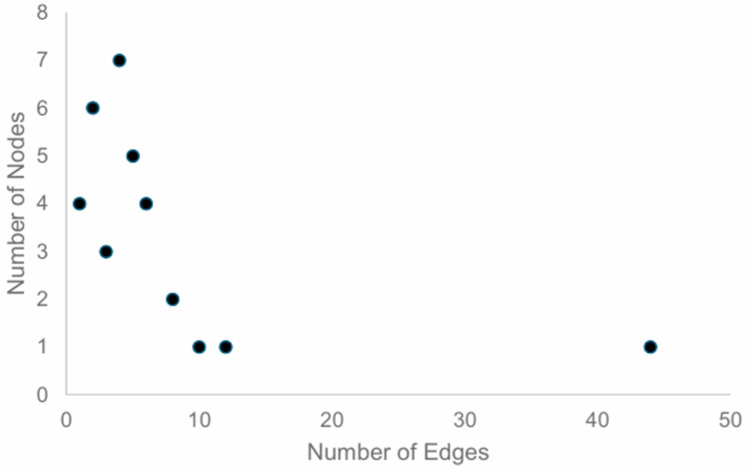
SKM034 node degree distribution. Number of nodes is shown on the *y*-axis, whilst the number of edges is shown on the *x*-axis. As an example of how to interpret the chart, the data point on the far right of the graph represents the fact that only 1 node (*y*-axis value) has more than 40 interactions (*x*-axis). In this situation, the node in question is SORL1.

The node degree distribution for the SKM034 model can be seen in Figure 3.

The node degree distribution is helpful in assessing which nodes to perform in silico knockouts on; the most connected nodes are the ones most likely to have an impact on the network if they are knocked out. For the SKM034 model, as shown in the node degree distribution, only one node (SORL1) exhibited an edge count > 40 at 44 edges. The most connected nodes after SORL1 were APP (12 edges), BACE1 (10 edges), ERBB2 (8 edges), and IL6 (8 edges).

Following this initial visualisation and analysis of basic model characteristics, the model was imported into CellNetAnalyzer (CNA) for both logical steady state analysis (LSSA) and dependency matrix (DM) analysis.

### 3.3. Dependency and in Silico Knockout Analysis of SKM034 Model

**Figure 4:**
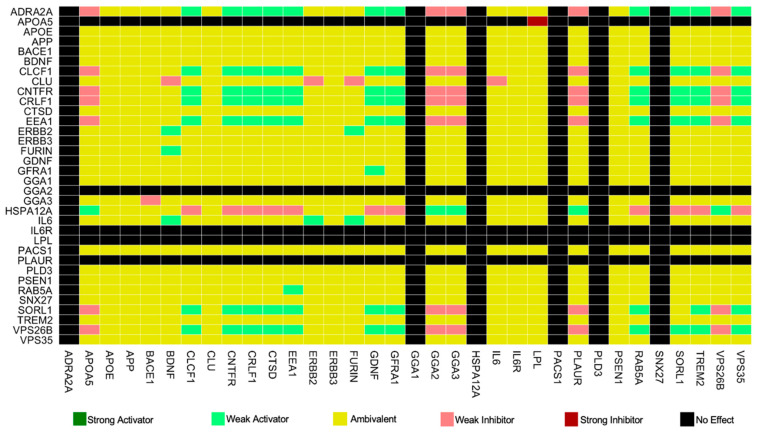
Dependency matrix for the wild-type SKM034 model. Dependencies show the effect of the node on the *y*-axis on the node on the *x*-axis. As summarised at the bottom of the figure, there are six possible types of relationship: strong activator (dark green), weak activator (light green), ambivalent factor (yellow), weak inhibitor (pink), strong inhibitor (red), and no effect (black).

As described in Section 2, CNA can generate dependency matrices that capture the overall effect of each node in the model on each node in the model, as shown in Figure 4.

**Figure 5:**
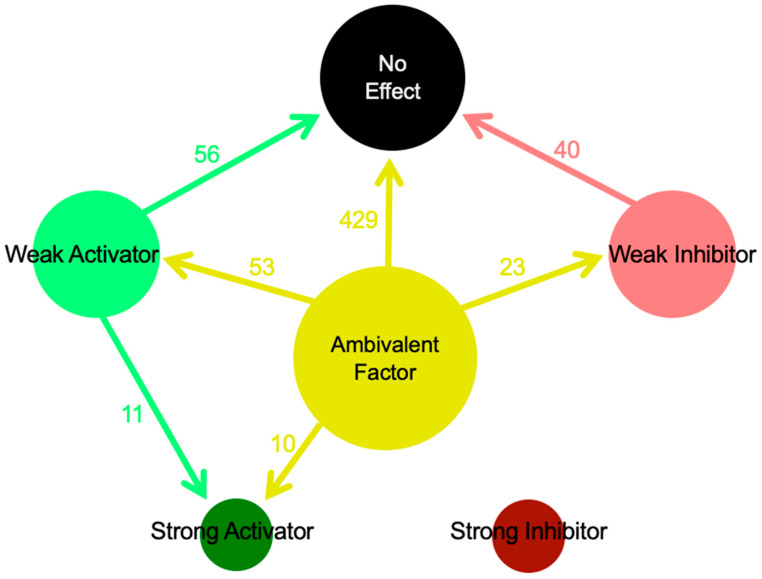
Dependency alteration distribution following an in silico SORL1 KO. The base scenario is the wild-type model, whilst the comparative scenario is SORL1 KO. This figure tracks the changes in specific relationships to other kinds of relationships. As an example, there were 10 dependencies that were ambivalent factors in the wild-type model. These 10 change to strong activators following SORL1 KO, thus representing the model’s prediction of how the molecular network is altered following perturbation.

In total, there are 1156 (34 × 34) dependencies in the full SKM034 model. Of these, 343 were no effect, 672 were ambivalent, 51 were weak inhibitors, 89 were weak activators, 1 was a strong inhibitor, and there were 0 strong activators. The majority of the dependencies being ambivalent represents a strong starting point for model analysis, as ambivalent dependencies are the ones most likely to change following an in silico knockout [12,13]. Additionally, changes to strong inhibitors or strong activators are the ones most likely to show an effect in vivo. The change in dependencies following an in silico SORL1 KO can be seen in Figure 5.

As shown in Figure 5, there were a total of 622 dependency changes following an in silico SORL1 KO. The most interesting changes are likely to be the 21 changes to strong activator relationships, with 10 of these coming from previously ambivalent relationships.

Based on the node degree distribution, additional knockouts were performed, which are summarised in Table 6.

It is important to note that the KO scenarios all have a total of 1089 dependencies (33 × 33) due to the removal of one node in each case. As highlighted previously, changes to strong activators or strong inhibitors are the ones most likely to show an effect in vivo. Table 7 below summarises all of these changes based on the knockout scenarios above, alongside literature validation (if any) and Potentially Novel Prediction (PNP) status.

As per Table 7, there are 29 changes to strong relationships. Literature validation demonstrates that 2 of these have been at least partially confirmed experimentally, and there are a total of 27 PNPs. Alongside the development of the model itself, these predictions represent a significant source of novelty in this manuscript, as they are clear routes forward for investigating SORL1 and its wider signalling.

### 3.4. Logical Steady State Analysis and Genome-Wide Model Validation (Cell Line Data)

To explore the SKM034 model further, logical steady state analysis (LSSA) was performed. In LSSA, all nodes are given an initial value of “Undetermined” (or NaN), and then inputs are manually set. To simulate SORL1 wild-type cells, SORL1 was set to 1 (ON), and then the logical steady state was computed through the signal flowing through the network. To simulate SORL1 KO cells, SORL1 was set to 0 (OFF), and the logical steady state was then computed. Comparison of the two scenarios permitted the calculation of *Emod* (i.e., the model’s prediction for the change of the node/gene). Table 8 shows the *Emod* values for all nodes in the network.

As evident in Table 8, the SKM034 model predicted that of the 33 nodes (other than SORL1, as it was manually set), 5 were upregulated in the SORL1 KO simulation, 10 were downregulated, and 18 were unchanged.

To validate these predictions based on experimental data, RNA-seq data from Lee and colleagues [18] was downloaded and analysed in a bespoke manner, as described in Section 2. The initial analysis, based solely on the *q*-value and the directionality of the fold-change, is shown in Table 9 alongside the *p*-value of the correct prediction rate.

As demonstrated in Table 9, there was a significant range in the correct prediction rates. Whilst this could be partially attributed to the relatively small size of the model (33 nodes being examined), meaning that small changes could lead to large differences, two other scenarios were hypothesised. The first was that the correct prediction rates would be correlated with the level of SORL1 knockdown, as this was noted to vary between the different cell types. The second was that adopting different degrees of biological change between the two conditions would lead to altered correct prediction rates, rather than simply whether the FC was <1 or >1.

**Figure 6:**
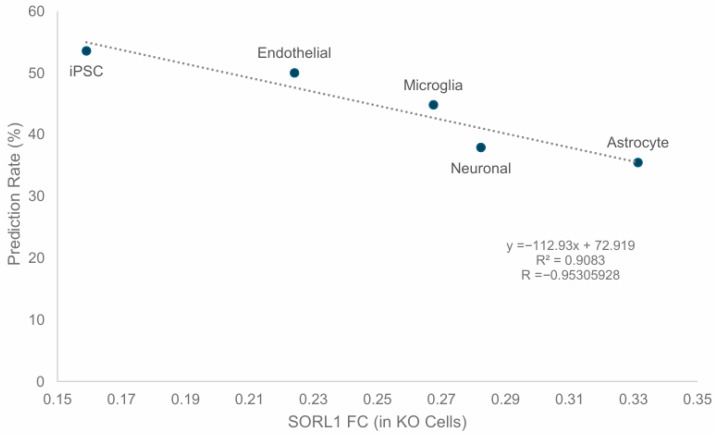
Correlation between level of SORL1 knockdown and model correct prediction rates. Relative amounts of SORL1 following knockdown are shown on the *x*-axis, whilst the *y*-axis shows the model’s prediction rate for that cell type. As shown, the prediction rate correlated strongly with the relative amount of SORL1, providing evidence for the first hypothesis described above.

The first hypothesis was investigated through a correlation analysis of SORL1 FC (indicating the level of SORL1 knockdown) against the correct prediction rates achieved, as shown in Figure 6.

Pearson’s R for the correlation between SORL1 FC and the correct prediction rate was −0.953, whilst the R^2^ was 0.908, indicating that 90.8% of the variation in the correct prediction rates was due to the level of SORL1 FC. Additionally, the *p*-value of the correlation was 0.032859988. Taken together, these provided strong evidence to support the first hypothesis stated above, but to explore the data further, validations were also conducted against FC 1.5, FC 2.0, and FC 3.0 (or reverse equivalent in all cases). This meant that if a gene had a *q*-value < 0.05, but was only marginally changed in its numerical value, it would instead be counted as “unchanged”. The results for these analyses are shown in Supplementary Tables S2 and S3.

As stated in Supplementary Tables S2 and S3, the correct prediction rates reached as high as 58.62%, demonstrating significant predictive utility relative to a random model. To extend the validation further beyond a molecular/cellular level, clinical validation in a cancer context was also performed.

### 3.5. Clinical Validation in Cancer Patients

In addition to the cell line validation above, the *Emod* values were compared to *Eexp* values generated from cancer patient data (as described in Section 2). The cBioPortal database [19,20,21] was used to access six brain cancer studies: Brain Lower Grade Glioma (TCGA, Firehose Legacy), Brain Lower Grade Glioma (TCGA, PanCancer Atlas), Brain Tumor PDXs (Mayo Clinic, Clin Cancer Res 2020), Glioblastoma (TCGA, Cell 2013), Glioblastoma Multiforme (TCGA, Firehose Legacy), and Glioblastoma Multiforme (TCGA, PanCancer Atlas). Similar to the cell line validation data, analysis was performed initially based on *q*-value and Log10 FC (Table 10). This analysis was later expanded to include a degree of biological change threshold (see Section 2 for a full explanation). These results can be seen in Table 10 (base values) and Supplementary Tables S4–S6.

As shown from Table 10 and Supplementary Tables S4–S6, the highest correct prediction rate was 60%, which demonstrates the significant predictive clinical utility of this Alzheimer’s-related model in a cancer context.

## 4. Discussion

This article employed established computational modelling approaches to investigate signalling mechanisms of SORL1, which is known to be important in both Alzheimer’s disease and cancer. The model developed, SKM034, demonstrates predictive utility as high as 60% at the clinical level, which in and of itself is an impressive feat given it is a small (34-node) model at the molecular level. In addition to the novelty of applying this modelling approach to SORL1 and assessing the interplay between Alzheimer’s disease and brain cancer, an additional novelty in this article is the use of bespoke analysis of cBioPortal data to validate these models in a clinical context.

This modelling approach, as previously described, has been employed in cancer contexts. This includes the previously published TP53 model (PKT206) [12], which was initially validated against cell line data from colon cancer and osteosarcoma and demonstrated correct prediction rates ranging from 52 to 71%. The same model was later integrated with mesothelioma patient data and permitted patient stratification and identification of potential survival-associated genes, as well as a range of putative repurposed drugs [14]. Whilst the TP53 model achieved significant predictive success and was utilised in multiple articles [14,22] following its initial publication [12], it is significantly larger than the SKM034 model (206 nodes versus 34 and 731 edges versus 92). As such, a more comparable model would be the model of the glucocorticoid receptor (GR, the GEB052 model) [13].

The GEB052 model consisted of 52 nodes, but only 49 of these were genes/proteins (the remainder were the inputs or outputs) [13]. The model exhibited a higher degree of interconnectivity than the SKM034 model, with 201 interactions between its 49 gene/protein nodes [13]. However, despite the GEB052 model being more resistant to perturbation (due to the higher number of feedback loops), correct prediction rates appear broadly comparable between it and the SKM034 model. Although the SKM034 model has a lower floor in its predictive capacity, the highest correct prediction rates for LSSA were comparable between the two models, at approximately 60% in both cases [13]. This is also confirmed in Supplementary Table S7, which statistically compares the two models and finds no significant difference in their correct prediction rate. Thus, the SKM034 model appears broadly comparable with previously published work.

It is interesting to note that two papers have previously explored the molecular and cellular association between AD and glioblastoma multiforme (GBM) [23,24]—work by Cai and colleagues [23], as well as work by Zhang and colleagues [24].

Cai and colleagues (2022) [23] explored the inverse association between GBM and AD through a bioinformatic approach using classic DEG and PPI network analysis. They analysed transcriptomic datasets from TCGA, GEO, and GTex, and identified 122 shared DEGs and 13 hub genes, and found AMPG, p53, and cell cycle regulation to be enriched pathways. Their focus, however, was on broad transcriptomic data available online, as well as pathway-level analysis, in contrast to the focus in this article on functional relationships that are changed following in silico knockouts.

Zhang and colleagues (2023) [24] also explored the connection between GBM and AD but focused on the role of microglia using single-cell RNA sequencing (scRNA-seq) and network analysis. They identified 11 microglia-mediated dysregulated genes shared by the two diseases, such as *BACE1* and *FURIN*, which are both involved in amyloid-β formation and tumour progression. Reassuringly, these were identified in our study and are part of the SKM034 model, which further validates their potential pathophysiological role. Zhang and colleagues focused on microglia and suggested a molecular link through cell-specific networks. This aligns with data presented here that also expands analysis to include other cell types, including iPSCs, astrocytes, neurons, and endothelial cells.

### Conclusions, Limitations, and Future Directions

Our paper investigated the hypothesis that there is a molecular link between glioma and AD that might contribute to the reported inverse correlation. This investigation was performed by analysing *SORL1*, a known AD risk gene, and its interacting genes and proteins. This was achieved by generating a SORL1 network of associated genes and proteins in an established two-layer workflow. Additionally, the SKM034 model established activation and inhibition relationships between SORL1 and its interacting genes and proteins. These enabled novel insight into new dysregulated genes in the SORL1 network and a model using cell transcriptomic data, and represent the first application of this modelling approach to investigate the potential interplay between AD and GBM.

As with any modelling approach, there are several limitations in this paper. One significant limitation of the analyses presented in this article is that they are Boolean (i.e., 1 or 0) and based on network topology. It has been previously recognised that such analyses are limited in a molecular signalling pathway context [13,22] as regulation of genes and proteins is not as simple as on or off but is instead dependent on (for example) variable levels of mRNA expression and protein production, as well as broader levels of control. To account for this, alternative analytical algorithms have historically been employed, such as the signal transduction score flow algorithm (STSFA) [25]. The advantage of the STSFA algorithm is that it allows for semi-quantitative analysis, and this has been shown to significantly boost the predictive power of Boolean models; for example, in the case of the GEB052 model, use of STSFA boosted predictive power to an average of 80.1% correct predictions, compared to a baseline of 56.6% from LSSA [13]. Thus, one area of future work for this model would be extensive semi-quantitative analyses. The model would also benefit from the addition of biological outputs, which could be identified through key resources such as DAVID [26] or Metascape [27]. This could further facilitate the development of experiments to build on the model’s predictions. Finally, as mentioned in Section 2, interaction records were only included if there was evidence in both a human and brain tissue context. This limited the number of interactions that were included, but also permitted greater focus on interactions known to occur and be relevant in the specific area of biology investigated. Future iterations of the model could consider expanding to incorporate non-human or non-brain evidence, which would increase the number of nodes and, potentially, the number of predictions, as well as predictive capacity.

Despite the limitations noted above, it is nonetheless evident that the SKM034 model represents a positive contribution to the understanding and clinical relevance of SORL1 and its interaction partners in both a brain cell and cancer context. With statistically significant correct prediction rates up to 60% (relative to a random model, which achieved a 33.3% correct prediction rate), the model is comparable to previously published models surrounding different genes/proteins. Additionally, the significant number of dependency matrix changes and predictions of strong relationships represent a solid foundation from which to undertake in vitro experimental work to verify the predictions and thus further accelerate research on this important protein and its wider network.

## Figures and Tables

**Table 1:** Filtration criteria used for Second Layer interaction PubMed searches. Column 1 of the table describes the approach at each filtering stage, whilst Column 2 shows exemplar replicable search terms. Filtration was not implemented until more than 60 papers were returned in the initial search result.

Search Terms	Filter Text
Date of Publication (Initial Search)	(((Protein 1)) AND ((Protein2))) AND ((“2023/07/01”[Date—Publication]: “2024/07/31”[Date—Publication]))
Filter Date of Publication, Title/Abstract	(((“2023/07/01”[Date—Publication]: “2024/07/31”[Date—Publication]))) AND ((((Protein1[Title/Abstract] OR Protein1[Title/Abstract] OR Protein2[Title/Abstract])) AND ((Protein2[Title/Abstract] OR Protein2[Title/Abstract]))))
Date Publication, Title	(((“2023/07/01”[Date—Publication]: “2024/07/31”[Date—Publication]))) AND ((((Protein1[Title] OR Protein1[Title] OR Protein1[Title])) AND ((Protein2[Title] OR Protein2[Title] OR Protein2[Title] Protein2[Title]))))

**Table 2:** *Emod* calculation. *Emod* shows the model’s prediction for how a node will change between two different scenarios and requires an LSS value from one scenario (Column 1) and an LSS value from a second scenario (Column 2). As shown in Column 3, *Emod* takes values of either −1, 0, or 1, referring to downregulated, unchanged, or upregulated, respectively (as described in Column 4).

LSS in Wild-Type Model	LSS in KO Model	*Emod*	Meaning
1	1	0	Unchanged
1	NaN	−1	Downregulated in mutant scenario
1	0	−1	Downregulated in mutant scenario
NaN	1	1	Upregulated in mutant scenario
NaN	NaN	0	Unchanged
NaN	0	−1	Downregulated in mutant scenario
0	1	1	Upregulated in mutant scenario
0	NaN	1	Upregulated in mutant scenario
0	0	0	Unchanged

**Table 3:** Possible dependencies in CNA. There are six different types of dependencies (Column 1), which are given different colours in the dependency matrix output (Column 2). Each dependency has specific requirements to arise, as summarised in Column 3.

Dependency of Node A on Node B	Colour on Dependency Matrix	Requirement
No Effect	Black	No paths present.
Ambivalent Factor	Yellow	Both positive and negative paths present.
Weak Inhibitor	Pink	There are negative paths from A to B and no positive paths. However, there is at least one negative feedback loop present in these negative paths.
Strong (Total) Inhibitor	Red	There are negative paths from A to B and no positive paths. There are also no negative feedback loops present in these negative paths.
Weak Activator	Light Green	There are positive paths from A to B and no negative paths. However, there is at least one negative feedback loop present in these positive paths.
Strong (Total) Activator	Dark Green	There are positive paths from A to B and no negative paths. There are also no negative feedback loops present in these positive paths.

**Table 4:** *Eexp* calculation for cell line validation. *Eexp* is used to show the change of a gene in real data (e.g., experimental data or clinical transcriptomic data) and can be compared with *Emod* to validate the model’s predictions. *Eexp* values are listed in Column 2, whilst Column 1 indicates how they could arise based on *q*-value and/or fold-change. Column 3 indicates the meaning of each *Eexp* value.

Situation	*Eexp*	Meaning
*q*-value > 0.05	0	Gene is unchanged in *SORL1*-KO cells
*q*-value < 0.05 and FC < 1	−1	Gene is downregulated in *SORL1*-KO cells
*q*-value < 0.05 and FC > 1	1	Gene is upregulated in *SORL1*-KO cells

**Table 5:** *Eexp* calculation for clinical validation. *Eexp* is used to show the change of a gene in real data (e.g., experimental data or clinical transcriptomic data) and can be compared with *Emod* to validate the model’s predictions. *Eexp* values are listed in Column 2, whilst Column 1 indicates how they could arise based on *q*-value and/or Log10 fold-change. Column 3 indicates the meaning of each *Eexp* value.

Situation	*Eexp*	Meaning
*q*-value > 0.05	0	Gene is unchanged in patients with low SORL1 expression
*q*-value < 0.05 and Log10 FC < 0	−1	Gene is downregulated in patients with low SORL1 expression
*q*-value < 0.05 and Log10 FC > 1	1	Gene is upregulated in patients with low SORL1 expression

**Table 6:** Summary of dependency matrix results following in silico knockouts. Column 1 describes the modelling scenario, whilst Columns 2–7 show the number of each type of dependency for that model. Column 8 shows the total number of dependencies for each modelling scenario.

Scenario	Number of Each Dependency						
	No Effect	Ambivalent	Weak Inhibitor	Weak Activator	Strong Inhibitor	Strong Activator	Total
**Full Model**	343	672	51	89	1	0	1156
**APP KO**	332	517	105	134	1	0	1089
**BACE1 KO**	359	433	106	190	1	0	1089
**ERBB2 KO**	430	369	114	167	4	5	1089
**IL6 KO**	359	438	118	173	1	0	1089
**SORL1 KO**	857	123	28	59	1	21	1089

**Table 7:** Summary of dependency changes to strong relationships, literature validation, and Potentially Novel Prediction (PNP) status. Column 1 shows the node that was deleted in the modelling scenario, whilst Column 2 and Column 3 represent the source node and target node, respectively. Column 4 and Column 5 show the relationships between the nodes in both the full (wild-type) model and the KO model. Column 6 shows the PubMed ID for papers that validate the relationship change predicted by the model. If no such literature could be found, Column 7 denotes it as a Potentially Novel Prediction (PNP).

Node Deleted	Node A	Node B	Original Relationship (Full Model)	New Relationship (KO Model)	Literature Validation	Novel Prediction?
SORL1	GDNF	GDNF	Ambivalent	Strong Activator	23333276	N/A—Prediction Fully Verified in Literature
SORL1	GDNF	GRFA1	Ambivalent	Strong Activator	N/A	N/A
SORL1	GRFA1	GRFA1	Ambivalent	Strong Activator	N/A	PNP
SORL1	GGA1	EEA1	Ambivalent	Strong Activator	N/A	PNP
SORL1	GGA1	RAB5A	Ambivalent	Strong Activator	N/A	PNP
SORL1	PACS1	GGA3	Ambivalent	Strong Activator	N/A	PNP
SORL1	TREM2	APOE	Ambivalent	Strong Activator	32941599	N/A—Prediction Fully Verified in Literature
SORL1	TREM2	CLU	Ambivalent	Strong Activator	N/A	PNP
SORL1	TREM2	CTSD	Ambivalent	Strong Activator	N/A	PNP
SORL1	VPS35	CTSD	Ambivalent	Strong Activator	N/A	PNP
SORL1	CLCF1	CLCF1	Weak Activator	Strong Activator	N/A	PNP
SORL1	CLCF1	CNTFR	Weak Activator	Strong Activator	N/A	PNP
SORL1	CLCF1	CRLF1	Weak Activator	Strong Activator	N/A	PNP
SORL1	CNTFR	CLCF1	Weak Activator	Strong Activator	N/A	PNP
SORL1	CNTFR	CNTFR	Weak Activator	Strong Activator	N/A	PNP
SORL1	CNTFR	CRLF1	Weak Activator	Strong Activator	N/A	PNP
SORL1	CRLF1	CLCF1	Weak Activator	Strong Activator	N/A	PNP
SORL1	CRLF1	CNTFR	Weak Activator	Strong Activator	N/A	PNP
SORL1	CRLF1	CRLF1	Weak Activator	Strong Activator	N/A	PNP
SORL1	GFRA1	GDNF	Weak Activator	Strong Activator	N/A	PNP
SORL1	RAB5A	EEA1	Weak Activator	Strong Activator	N/A	PNP
ERBB2	FURIN	BDNF	Weak Activator	Strong Activator	N/A	PNP
ERBB2	CLU	LPL	Ambivalent	Strong Activator	N/A	PNP
ERBB2	IL6	IL6R	Ambivalent	Strong Activator	N/A	N/A
ERBB2	PACS1	BDNF	Ambivalent	Strong Activator	N/A	PNP
ERBB2	PACS1	FURIN	Ambivalent	Strong Activator	N/A	PNP
ERBB2	CLU	IL6R	Ambivalent	Strong Inhibitor	N/A	PNP
ERBB2	IL6	LPL	Ambivalent	Strong Inhibitor	N/A	N/A
ERBB2	CLU	IL6	Weak Inhibitor	Strong Inhibitor	N/A	PNP

**Table 8:** *Emod* values for LSS analysis. Column 1 shows the node in question, whilst Columns 2 and 3 show the node’s LSS value for both wild-type (Column 2) and SORL1 KO (Column 3) scenarios. Column 4 shows the *Emod* value, calculated as described in Section 2.

Node	SORL1 WT Simulation (SORL1 = 1)	SORL1 KO Simulation (SORL1 = 0)	*Emod*
ADRA2A	NaN	NaN	0
APOA5	0	1	1
APOE	1	1	0
APP	1	1	0
BACE1	1	1	0
BDNF	1	1	0
CLCF1	1	NaN	−1
CLU	1	1	0
CNTFR	1	NaN	−1
CRLF1	1	NaN	−1
CTSD	1	0	−1
EEA1	1	NaN	−1
ERBB2	1	1	0
ERBB3	1	1	0
FURIN	1	1	0
GDNF	1	NaN	−1
GFRA1	1	NaN	−1
GGA1	NaN	NaN	0
GGA2	0	1	1
GGA3	NaN	1	1
HSPA12A	NaN	NaN	0
IL6	1	1	0
IL6R	1	1	0
LPL	1	1	0
PACS1	NaN	NaN	0
PLAUR	0	1	1
PLD3	NaN	NaN	0
PSEN1	1	1	0
RAB5A	1	NaN	−1
SNX27	NaN	NaN	0
SORL1	1	0	N/A (manually set)
TREM2	1	0	−1
VPS26B	0	1	1
VPS35	1	0	−1

**Table 9:** Correct prediction rates for the SKM034 model based on the data from Lee and colleagues [18]. Statistically significant *p*-values (by conventional criteria *p* < 0.05) are emphasised in bold. Column 1 shows the Cell Type, whilst Column 2 shows the model’s correct prediction rate. Column 3 shows the *p*-value of the correct prediction rate.

Cell Type	Correct Prediction Rate (%)	*p*-Value of Correct Predictions
iPSC	53.57	**0.013407744**
Neuronal	37.93	0.132149528
Astrocyte	35.48	0.143741592
Microglia	44.83	0.064804096
Endothelial	50.00	**0.024687275**

**Table 10:** Summary of correct prediction rates (%) from mRNA z-scores diploid to samples in patient RNA-seq data. The study names from cBioPortal are shown in Column 1, whilst different analytical thresholds are shown in Columns 2–5.

Study	Z-Scores by Mean Correct Prediction Rate (%)	Z-Scores by Median Correct Prediction Rate (%)	Z-Scores by Upper Quartile Correct Prediction Rate (%)	Z-Scores by Lower Quartile Correct Prediction Rate (%)
Brain Lower Grade Glioma (TCGA, Firehose Legacy)	36.36	36.36	45.45	30.30
Brain Lower Grade Glioma (TCGA, PanCancer Atlas)	45.45	36.36	48.48	33.33
Brain Tumor PDXs (Mayo Clinic, Clin Cancer Res 2020)	54.55	54.55	54.55	54.55
Glioblastoma (TCGA, Cell 2013)	54.55	54.55	36.36	30.3
Glioblastoma Multiforme (TCGA, Firehose Legacy)	45.45	39.39	45.45	51.52
Glioblastoma Multiforme (TCGA, PanCancer Atlas)	51.52	42.42	48.48	48.48

## Data Availability

The original contributions presented in this study are included in the article/Supplementary Materials. Further inquiries can be directed to the corresponding authors.

## Supplementary Materials

The following supporting information can be downloaded at: https://www.mdpi.com/article/10.3390/cimb48020126/s1.

## Abbreviations

The following abbreviations are used in this manuscript:

AD	Alzheimer’s Disease
Aβ peptide	Amyloid-beta Peptide
CNA	CellNetAnalyzer
DMs	Dependency Matrices
FC	Fold Change
GBM	Glioblastoma Multiforme
GR	Glucocorticoid Receptor
iPSCs	Induced Pluripotent Stem Cells
LSSA	Logical Steady State Analysis
NaN	Undetermined (Not A Number)
PNPs	Potentially Novel Predictions
scRNA-seq	single-cell RNA sequencing
SKM034	SORL1 model by Kristy Montalbo, consisting of 34 nodes
STRING	Search Tool for the Retrieval of Interacting Genes/Proteins
