# Supplementary material for: Computational Modelling and Clinical Validation of an Alzheimer’s-Related Network in Brain Cancer: The SKM034 Model
Source: Curr Issues Mol Biol. 2026 Jan 23;48(2):126. doi: 10.3390/cimb48020126 (PMC12939318; doi:10.3390/cimb48020126)
Supplement: Supplementary file 1 [file cimb-48-00126-s001.zip › cimb-4083017-supplementary.pdf]

**Montalbo et al. Supplementary File S1**

**Supplementary Table S1A.** Primary Layer Interactions

| Protein 1 | Activates (1) or Inhibits (0) | Protein 2 | PubMed ID |
|---|---|---|---|
| ADRA2A | 1 | SORL1 | 25404298 |
| APOE | 0 | SORL1 | 28634550 |
| BDNF | 1 | SORL1 | 25772071 |
| CLCF1 | 1 | SORL1 | 28117780 |
| CRLF1 | 1 | SORL1 | 28117780 |
| EEA1 | 1 | SORL1 | 35226190 |
| ERBB2 | 1 | SORL1 | 34564954 |
| ERBB3 | 1 | SORL1 | 33420373 |
| FURIN | 1 | SORL1 | 11294867 |
| GFRA1 | 1 | SORL1 | 23333276 |
| GGA1 | 0 | SORL1 | 22621900 |
| HSPA12A | 0 | SORL1 | 30679749 |
| PACS1 | 0 | SORL1 | 24001769 |
| PLD3 | 0 | SORL1 | 29368044 |
| PSEN1 | 0 | SORL1 | 16930450 |
| RAB5A | 1 | SORL1 | 34133918 |
| SNX27 | 1 | SORL1 | 27466343 |
| SORL1 | 1 | APP | 35226190 |
| SORL1 | 0 | APOE | 37611586 |
| SORL1 | 0 | GGA2 | 22621900 |
| SORL1 | 1 | VPS35 | 35226190 |
| SORL1 | 0 | CLU | 37611586 |
| SORL1 | 1 | PSEN1 | 19036982 |
| SORL1 | 0 | BACE1 | 16407538 |
| SORL1 | 1 | TREM2 | 34785643 |
| SORL1 | 0 | LPL | 21385844 |
| SORL1 | 0 | IL6 | 28265003 |
| SORL1 | 1 | GFRA1 | 23333276 |
| SORL1 | 0 | PLAUR | 15053742 |
| SORL1 | 1 | CTSD | 34133918 |
| SORL1 | 0 | VPS26B | 34965419 |
| SORL1 | 1 | GDNF | 21994944 |
| SORL1 | 1 | CRLF1 | 20584990 |
| SORL1 | 0 | GGA3 | 22762444 |
| SORL1 | 0 | APOA5 | 17326667 |
| SORL1 | 1 | CNTFR | 28117780 |
| SORL1 | 1 | RAB5A | 32492427 |

| | | | |
|---|---|---|---|
| SORL1 | 0 | IL6R | 28265003 |
| SORL1 | 1 | CLCF1 | 20584990 |
| SORL1 | 1 | ERBB2 | 31138794 |
| SORL1 | 1 | ERBB3 | 33420373 |
| TREM2 | 1 | SORL1 | 34785643 |
| VPS26B | 1 | SORL1 | 34965419 |
| VPS35 | 0 | SORL1 | 17646382 |

**Supplementary Table S1B.** Second Layer Interactions

| Protein 1 | Activates (1) or Inhibits (0) | Protein 2 | PubMed ID |
|---|---|---|---|
| APOA5 | 0 | LPL | 38625948 supported by 37824203 |
| APOE | 1 | APP | 9593736 supported by 33148290 |
| APP | 0 | BACE1 | 28382744 |
| APP | 1 | IL6 | 33013850 |
| BACE1 | 1 | APP | 10531052 |
| BACE1 | 1 | PSEN1 | 12901838 |
| BACE1 | 0 | IL6 | 3429071 |
| BDNF | 1 | APP | 28382744 |
| BDNF | 0 | IL6 | 35009001 |
| BDNF | 1 | ERBB2 | 28446206 |
| BDNF | 0 | BACE1 | 26293123 |
| CLCF1 | 1 | CRLF1 | 10966616 supported by 31552057 |
| CLCF1 | 1 | CNTFR | 10966616 supported by 11285233 |
| CLU | 0 | IL6 | 35705674 |
| CNTFR | 1 | CLCF1 | 11285233 |
| CNTFR | 1 | CRLF1 | 26858303 supported by 10966616 |
| CRLF1 | 1 | CLCF1 | 10966616 supported by 27903646 |
| CRLF1 | 1 | CNTFR | 26858303 supported by 10966616 |
| CTSD | 1 | APP | 26002056 supported by 10605825, 9236226, 7523115 |
| CTSD | 1 | ERBB2 | 32846884 |
| ERBB2 | 1 | ERBB3 | 34171264 |
| ERBB2 | 1 | FURIN | 32029900 |
| ERBB3 | 1 | ERBB2 | 34171264 |
| FURIN | 1 | BACE1 | 10956649 |
| FURIN | 0 | LPL | 30021841 supported by 16109723 |
| FURIN | 1 | BDNF | 31558607 |
| GDNF | 1 | GFRA1 | 32985758 |
| GDNF | 1 | PSEN1 | 10473269 |
| GFRA1 | 1 | GDNF | 32985758 |
| GGA1 | 0 | BACE1 | 26053850 supported by 15466887 |
| GGA1 | 0 | APP | 26053850 |
| GGA1 | 1 | RAB5A | 12505986 |
| GGA3 | 0 | BACE1 | 17553422 |
| IL6 | 1 | IL6R | 38441314 |
| IL6 | 1 | ERBB2 | 9590694 |

| | | | |
|---|---|---|---|
| IL6 | 0 | LPL | 15531514 |
| PACS1 | 1 | FURIN | 1360148 |
| PACS1 | 1 | GGA3 | 16977309 |
| PLD3 | 0 | APP | 24336208 |
| PSEN1 | 1 | APP | 30980041 supported by 9223340 |
| RAB5A | 1 | EEA1 | 37725090 |
| RAB5A | 1 | BACE1 | 17325690 |
| SNX27 | 0 | APP | 25437537 |
| TREM2 | 1 | APOE | 27477018 |
| TREM2 | 1 | CLU | 27477018 |
| TREM2 | 1 | CTSD | 34301296 |
| VPS35 | 1 | CTSD | 24152121 supported by 35045281, 28722658 |
| VPS35 | 0 | APP | 22516235 supported by 25745458, 22105352 |

**Supplementary Table S2.** SKM034 model validation using the RNA-seq data from Lee and colleagues [18] based on different biological thresholds. In this table, mean values were calculated for each gene based on the replicate samples.

| Cell Line | Mean FC 1.5 or Equivalent Correct Prediction Rate (%) | Mean FC 2 or Equivalent Correct Prediction Rate (%) | Mean FC 3 or Equivalent Correct Prediction Rate (%) |
|---|---|---|---|
| iPSC | 57.14 | 57.14 | 57.14 |
| Neuronal | 44.83 | 55.17 | 48.28 |
| Astrocyte | 45.16 | 48.39 | 51.61 |
| Microglia | 48.28 | 48.28 | 48.28 |
| Endothelial | 53.33 | 54.84 | 53.33 |

**Supplementary Table S3.** SKM034 model validation using the RNA-seq data from Lee and colleagues [18] based on different biological thresholds. In this table, median values were calculated for each gene based on the replicate samples.

| Cell Line | Median FC 1.5 or Equivalent Correct Prediction Rate (%) | Median FC 2 or Equivalent Correct Prediction Rate (%) | Median FC 3 or Equivalent Correct Prediction Rate (%) |
|---|---|---|---|
| iPSC | 55.17 | 58.62 | 58.62 |
| Neuronal | 37.93 | 44.83 | 48.28 |
| Astrocyte | 45.16 | 48.39 | 51.61 |
| Microglia | 48.28 | 48.28 | 48.28 |
| Endothelial | 53.33 | 53.33 | 53.33 |

**Supplementary Table S4.** Summary of correct prediction rates (%) for FC 1.5 or equivalent of raw mRNA expression. Note that there are fewer studies included here than in Table 10 due to limited availability of study data.

| | FC 1.5 or Equivalent | | | |
|---|---|---|---|---|
| Study | Raw by Mean Correct Prediction Rate (%) | Raw by Median Correct Prediction Rate (%) | Raw by Upper Quartile Correct Prediction Rate (%) | Raw by Lower Quartile Correct Prediction Rate (%) |

| | | | | |
|---|---|---|---|---|
| Brain Lower Grade Glioma (TCGA, Firehose Legacy) | 57.58 | 57.58 | 48.48 | 45.45 |
| Glioblastoma (TCGA, Cell 2013) | 36.36 | 42.42 | 45.45 | 30.30 |
| Glioblastoma Multiforme (TCGA, Firehose Legacy) | 48.48 | 48.48 | 48.48 | 48.48 |

**Supplementary Table S5.** Summary of correct prediction rates (%) for FC 2 or equivalent of raw mRNA expression. Note that there are fewer studies included here than in Table 10 due to limited availability of study data.

| | **FC 2 or Equivalent** | | | |
|---|---|---|---|---|
| **Study** | **Raw by Mean Correct Prediction Rate (%)** | **Raw by Median Correct Prediction Rate (%)** | **Raw by Upper Quartile Correct Prediction Rate (%)** | **Raw by Lower Quartile Correct Prediction Rate (%)** |
| Brain Lower Grade Glioma (TCGA, Firehose Legacy) | 54.55 | 54.55 | 60.00 | 57.58 |
| Glioblastoma (TCGA, Cell 2013) | 48.48 | 48.48 | 51.52 | 39.39 |
| Glioblastoma Multiforme (TCGA, Firehose Legacy) | 54.55 | 54.55 | 54.55 | 54.55 |

**Supplementary Table S6.** Summary of correct prediction rates (%) for FC 3 or equivalent of raw mRNA expression. Note that there are fewer studies included here than in Table 10 due to limited availability of study data.

| | **FC 3 or Equivalent** | | | |
|---|---|---|---|---|
| **Study** | **Raw by Mean Correct Prediction Rate (%)** | **Raw by Median Correct Prediction Rate (%)** | **Raw by Upper Quartile Correct Prediction Rate (%)** | **Raw by Lower Quartile Correct Prediction Rate (%)** |
| Brain Lower Grade Glioma (TCGA, Firehose Legacy) | 54.55 | 54.55 | 60.00 | 57.58 |
| Glioblastoma (TCGA, Cell 2013) | 54.55 | 54.55 | 54.55 | 48.48 |
| Glioblastoma Multiforme (TCGA, Firehose Legacy) | 54.55 | 54.55 | 54.55 | 54.55 |

**Supplementary Table S7.** Comparison of correct prediction rates for GEB052 and SKM034. Note that the correct prediction rates for GEB052 were extracted from Bakker et al 2017 (https://journals.plos.org/ploscompbiol/article?id=10.1371/journal.pcbi.1005825#sec002). The top 5 values were taken. Similarly, for SKM034, the top 5 LSSA values from cell line data were taken from Supplementary Table 2, "Mean FC 2 or Equivalent Correct Prediction Rate (%)". P-value is determined by a two-sample, two-tailed, heteroscedastic t-test.

| **Model** | **GEB052** | **SKM034** |
|---|---|---|
| **Top 5 LSSA Correct Predictions** | 60.4 | 57.14 |
| | 58.3 | 55.17 |
| | 58.3 | 48.39 |
| | 54.2 | 48.28 |
| | 54.2 | 54.84 |
| **Average** | 57.08 | 52.764 |
| **STDEV** | 2.765320958 | 4.137817057 |
| **P-Value** | 0.093774997 | |
